# Standards of practice for hospital libraries and librarians, 2022: Medical Library Association Hospital Libraries Caucus Standards Task Force

**DOI:** 10.5195/jmla.2022.1590

**Published:** 2022-10-01

**Authors:** Jill Tarabula, Donna S. Gibson, Bridget Jivanelli, J. Michael Lindsay, Ana Macias, Sondhaya McGowan, Lori Mills, Louise McLaughlin

**Affiliations:** 1 jtarabula@cvph.org, Regional Medical Librarian, Champlain Valley Physicians Hospital (UVMHN), Plattsburgh, NY.; 2 GibsonD@mskcc.org, Memorial Sloan Kettering Cancer Center, New York, NY.; 3 jivanellib@hss.edu, Kim Barrett Memorial Library, HSS Education Institute, Hospital for Special Surgery, New York, NY.; 4 JMLindsay@utmck.edu, Associate Professor, Head of Collections and Access Services, Health Information Center, Preston Medical Library, UT Graduate School of Medicine, and UT Health Science Center, Knoxville, TN; 5 Ana.m.macias@kp.org, Manager Library Services, Kaiser Permanente; 6 Mcgowan.sunny@scrippshealth.org, Scripps Mercy Hospital, San Diego, CA.; 7 lori.mills@mclaren.org, Librarian, IPCE Coordinator, McLaren Macomb, Mt Clemens, MI.; 8 louise.mclaughlin@womans.org, Health Sciences Library (retired), Woman's Hospital, Baton Rouge, LA.

**Keywords:** Librarians, libraries, hospital library standards, professional role, library services/standards, personnel staffing and scheduling/standards, evidence-based information, virtual library services

## Abstract

The Hospital Library Caucus of the Medical Library Association (MLA) follows the practice established in 1953 of developing quality indicators and best practices in the newly developing and fast-changing world of hospital libraries. As these libraries increased in number and prominence, the Joint Commission on the Accreditation of Hospitals (JCAHO) included in 1978 a hospital library standard developed in collaboration with MLA. Subsequent changes in JCAHO, then The Joint Commission (TJC) knowledge management criteria as well as technological changes in the curation and delivery of evidence-based resources influenced standards changes over the years. The 2022 standards mark the most recent edition, replacing the 2007 standards.

## BACKGROUND

The Medical Library Association's (MLA's) “Standards for Hospital Libraries” were developed in 2002 as a guide for hospital administrators, librarians, and accrediting bodies to ensure that hospitals have the resources and services to effectively meet their needs for knowledge-based information (KBI) [1]. In approving the original and subsequent revised versions of the standards, the MLA Board of Directors recommended that the Standards Committee of the Hospital Libraries Section continually evaluate the standards and revise them as necessary to reflect changes in the health care environment and MLA priorities. This document reflects the most recent update of these standards.

## EXECUTIVE SUMMARY

MLA's “Standards of Practice for Hospital Libraries and Librarians, 2022” have been revised to reflect the technological, environmental, and budgetary changes so visible in hospitals today, with updates to meet the dynamic needs of 21^st^ century hospital libraries and the institutions they serve. The document serves a two-fold purpose:

- as a guide for hospital senior and department-level leaders, librarians, information technology (IT) teams, and accrediting bodies to ensure that libraries have the resources and services to effectively meet the hospital's KBI needs, to support evidence-based practices, quality and safety program development, and improved patient outcomes.
- as an organizational benchmark to be used to meet the KBI needs for continuous access to point of care resources, to improve access to documented best practices that improve patient outcomes and contribute to quality and performance improvement initiatives, while being mindful of budget, space, and staffing constraints.

While many of the library practices remain constant over the years, the Committee updated or added content to

- demonstrate the commitment to diversity, equity, and inclusion in all health care environments.
- create consistency through the use of the broader term “hospital” or “health sciences library” in place of “medical library.”
- bring to light the increasing involvement of librarians with IT applications and teams, including KBI source links in electronic health records (EHR), mobile application support, and interactions with electronic resource products and vendors.
- broaden the concept of library space to include physical and virtual libraries.
- highlight new research available for calculating staffing ratios and coordinating staffing with library service levels.
- expand roles in teaching, researching, and appraising evidence-based literature across clinical specialties and to hospital staff at every level.
- emphasize the significance of the librarian having an advanced degree and AHIP certification
- expand suggested duties of the librarian to include
  - support for publishing and adhering to copyright laws.
  - developing a needs assessment and strategic plan for the library.
  - planning physical and virtual library spaces including an intra- or internet presence.
- reference additional materials to support these recommended, evidence-based library practices.

The 2022 “MLA Statement Calling on Hospital and Health System Executives to Fund Libraries and Library Staff,” co-signed by multiple credentialing and health care institutions, adds additional support to the maintenance of library services. The health sciences librarian is positioned to play a key role in the hospital. The ubiquitous nature of the internet and primarily online collections, existing and disruptive technologies, and evolving means of communication by medical, nursing, and allied health staffs, patients, and the community require new strategies, strategic planning, allocation of adequate resources, and selection and evaluation of appropriate information resources and technologies. It is the hope of the Hospital Library Standards Committee that the material herein advances these efforts.

## SYNOPSIS OF STANDARDS OF PRACTICE

**Standard 1**: The librarian serves as the primary department head responsible for managing resources and services to meet the KBI needs of the organization. The library has its own budget, and the library director/manager, as a department head, reports to the senior management of the organization.

**Standard 2**: Health care research and reference information systems and services are directed by a qualified librarian. Academy of Health Information Professionals (AHIP) membership is preferred.

**Standard 3**: Informed by MLA's commitment to fostering diversity, equity, and inclusion, library staffing formulas and service level agreements guide human resource allocation.

**Standard 4**: The health sciences librarian, as the principal KBI professional in the organization, collaborates with the information technology team to ensure online resources and services are functional and available at the point of need, including in the EHR.

**Standard 5:** Evidence provides the scientific basis linking KBI and improved patient care; enhanced patient education; support for performance improvement projects; patient safety functions; and student, nursing, medical, graduate, and continuing medical education.

**Standard 6**: The librarian provides evidence of an ongoing assessment of the information needs of the organization, and the development and implementation of a plan to provide appropriate resources, services, and technology to meet those identified needs.

**Standard 7:** The librarian actively promotes KBI services and resources to all user groups and provides documented evidence thereof.

**Standard 8**: All KBI functions are performed in compliance with applicable federal, state, and local laws and regulations including but not limited to copyright, HIPAA (patient privacy and confidentiality), and vendor licensing agreements.

**Standard 9**: KBI resources are available to staff twenty-four hours a day, seven days a week.

**Standard 10**: The term library refers to the department—physical, virtual or a combination thereof—and includes a qualified librarian and support professionals, services, and resources that when put together, best meet the information needs of the health care organization.

**Standard 11**: IT resources are available to support the library's mission of providing KBI resources and services.

## INTRODUCTION

Following the publication of the Standards in 2002, a revised version was published in 2005 [1, 2]. In March of 2005, the National Network of Libraries of Medicine (NNLM) Hospital Internet Access Task Force issued a final report and made several recommendations, one of which was to work with the HLS Standards Committee to add a technology standard to the “Standards for Hospital Libraries.” The new standard defined the minimum levels of technology needed for hospital libraries to function in their role as providers of KBI resources. In May 2007, Standard 11 was approved by the MLA Board at its annual meeting. As well, the MLA educational policy statement and the *Competencies for Lifelong Learning and Professional Success* were added to the Standards document. Other pertinent updates were made to reflect changes in resources and terminology in use. Additionally, the MLA Board recommended that the revised standards be called “Standards for Hospital Libraries 2007” and that the publication history be indicated [3].

In October 2019, a discussion ensued regarding a review and update of the existing “Standards for Hospital Libraries 2007.” The Hospital Libraries Task Force was formed, with members volunteering to review specific standards for currency and application to modern practices and technologies. Despite COVID-19's impact and after numerous virtual meetings, a final draft was prepared for presentation to the Hospital Library Caucus in the Spring of 2022.

## DETAILED STANDARDS OF PRACTICE AND INTENT

**Standard 1:** The librarian serves as the primary department head responsible for managing resources and services to meet the KBI needs of the organization. The library has its own budget, and the library director/manager, as a department head, reports to the senior management of the organization [3, 4].

**Intent.** Library as department. As reflected in Joint Commission resources, access to KBI is an information management requirement for a hospital or health system [5–7]. To enable the development of systems, resources, and services to meet this requirement, the needs, concerns, and contribution of the library must be communicated to decision makers at the highest levels in the organization. Departmental status with the librarian in a director/manager level leadership position facilitates this connection. Librarian interaction with other departmental leaders and administrators fosters a deeper understanding of the information needs of the organization. This interaction can also facilitate access to institutional resources and data necessary for providing information to satisfy the needs of hospital senior leaders, clinicians, patients, and family members [8].

In 2022, the MLA issued a statement adding additional support to the provision and maintenance of curated library services.

[Excerpt] “Hospital and health system executives must take concrete action to support clinical and administrative staff, patients and their families, and communities by funding hospital library services led by a professional librarian. Working together, hospitals, health care systems, and librarians are positioned to provide the resources, access, and support your health care teams can depend upon to provide evidence-based practice services that will support healthy patient outcomes.”

National associations and institutions have endorsed the full statement supporting hospital library departments [9].

**Standard 2:** A qualified librarian directs medical research and reference information systems and services. Academy of Health Information Professionals membership is preferred.

**Intent.** Librarian qualifications. Providing medical, nursing, and clinical information requires specific knowledge and skills related to understanding information management and technologies, meeting rapidly changing user needs in a health care setting and provision of information services and resources aligning with the organization's mission, vision, goals, and strategic plan. Reliance on a commercial electronic resource for clinical information cannot substitute for a qualified medical librarian.

A qualified librarian is a person who has earned a Master's degree from a:

- program accredited by the American Library Association (ALA) or its successor accrediting organization [10] or
- program in library and information studies accredited or recognized by the appropriate national body of another country

As a health information professional, the health sciences librarian possesses unique knowledge, skills and abilities that have been identified as professional competencies. Evidence of continued advancement of these competencies is membership in MLA's Academy of Health Information Professionals (AHIP) [11]. The MLA has developed *Competencies for Lifelong Learning and Professional Success* outlining the essential professional skills and abilities that are expected of health sciences librarians. Competency areas include information services and management, instruction, leadership, evidence-based practice, research, and professionalism [12].

Provision of health information occurs in a variety of settings including hospitals and health care centers, research, and academic/medical, nursing, and allied health schools. Library and information services are developed that meet the strategic information needs of the individual or group being served. The role of the health sciences librarian may include but is not limited to [13–14]:

- developing, providing and promoting library services to support access to biomedical literature including:
  - mediated literature searches
  - document delivery/interlibrary loan (ILL)
  - current awareness services
- responding to consumer health requests related to patient care, health and disease, medications, and community resources
- selecting, evaluating, licensing/purchasing and promoting resources for incorporation into the physical or electronic collections
- development and maintenance of an online presence, to include remote access
- individual and institutional support for copyright compliance and publication
- providing new/existing employee orientation to resources and services
- strategic planning for library operations to support the mission of the institution
- budgeting for library operations; identifying partnerships and consortia purchasing opportunities
- hiring, training, and evaluating the performance of the library staff
- evaluating new technologies and assessing their application to library management and services
- physical space planning for the library

These roles and services are explored further in the following standards.

**Standard 3:** MLA's commitment to diversity, equity, and inclusion should form the foundation for determining staffing levels and service level agreements.

MLA fosters excellence and is committed to diversity, equity, and inclusion in professional practice, leadership of health sciences libraries, and for information professionals, now and in the future [15].

**Intent.** Staffing. An adequately staffed library is necessary to fully serve the KBI needs of the total institutional staff. Consideration for library staffing needs to include an understanding of the user community's information needs, new and potential users, and outreach endeavors that utilize/require library services. Library operations and workload are driven by the staff size, clinical research activities, and mission and complexity of the institution.

As stated in Standard 1, interaction of the librarian with other departmental leaders and administrators fosters a deeper understanding of the information needs of the organization. Working together, librarians, administrators, and stakeholders should decide upon the levels of library services when formulating a service level agreement.

We recommend that each hospital library utilize the grid in Table 1 presented in the Canadian Health Libraries Association (CHLA) Standards that considers Van Moorsel's “golden ratio,” MLA's 2007 Standards, and the chart developed by the Health Science Information Consortium of Toronto (HSICT) [16, 17, 3, 18]. The latter serves as a guide to outline possible services based on bronze, silver, and gold levels [Appendix A]. Attention must be called to Van Moorsel's use of the “mean” number (2,865) of FTE's as calculated from his survey results. Calibration using this method for gauging staffing requirements for relatively large or relatively small libraries must be carefully considered [18]. Updates to both Canadian works are in the planning stage.

**Table 1:** Staffing grid by HSICT level of service

Number of institutional staff*		Number of FTE health information professionals		
Staff	Sq. Root†	Bronze	Silver	Gold
400	20	1.24	1.55	1.85
625	25	1.55	1.93	2.32
900	30	1.85	2.32	2.78
1225	35	2.16	2.70	3.24
1600	40	2.47	3.09	3.71
2025	45	2.78	3.48	4.17
2500	50	3.09	3.86	4.63
3025	55	3.40	4.25	5.10
3600	60	3.71	4.63	5.56
4225	65	4.08	5.02	6.03
4900	70	4.33	5.41	6.49
5625	75	4.63	5.79	6.95
6400	80	4.94	6.18	7.42

* Includes all active medical staff, as well as health care personnel on service contract

† Calculated using square roots in increments of 5 from 20 to 80 (20x20=400; 25x25=625, etc.)

Future benchmarking studies could dive deeper by collecting specific data that would correlating the number of staff support per individual library service. The 2019 study by Spencer, Mamo, and Billman published in February 2021 is a step in this direction [19].

The amount of staffing throughout the system should be at least at the level specified in the library staffing formula for minimum level of service (bronze or silver), taking all components and needs of the health care system into account. Whether each hospital or institution is treated separately in determining staffing levels, or the system is taken as a whole, is left to the judgment of the health information professional and administrators. It stands to reason that librarians providing services at the gold level, such as support for systematic reviews and other knowledge synthesis, or research data management, etc. (or higher in the case of libraries providing services not listed in the HSICT document), will require additional staff. The important point is that staffing is sufficient to serve the number of users and is appropriate for the level of service required to meet the needs of the organization. The grid in Table 1 may be used to determine appropriate staffing needed by level of service [16].

The following formulae are used to calculate number of library staff needed per level of service:

Bronze √total FTE institution / 10 (1.61803399) [1.0 (16.180339) is an accurate alternative.]

Silver √total FTE institution x 1.25 / 16.1803399

Gold √total FTE institution x 1.5 / 16.1803399

Additional staffing is necessary if the librarian provides enhanced services (e.g., at the silver or gold levels) or services usually performed by other departments, or supports entities other than just a hospital, such as, but not limited to, the examples listed below:

- research data management
- publication and institutional repository support
- systematic review support
- primary responsibility for audiovisual equipment and other information technology
- substantial responsibility for web programming, hospital-wide intranet or internet site
- coordination of, or clerical support for, continuing medical education program
- networked system with multiple libraries
- support of entities other than hospital (e.g., research institutes, education programs)

This information provides a starting point for determining what service level is desired and what staffing will be required to support those needs at each institution [Appendix A].

In establishing library staffing size, consideration must also be given to the service level, hours of operations, and expectations agreed upon by the hospital administrator/hospital leadership team, stakeholders, and library director or information professional. Just having a staffing formula is not enough to accurately determine how best to serve the organization.

**Standard 4:** The health sciences librarian, as the principal KBI professional in the organization, collaborates with the information technology team to ensure online resources and services are functional and available at the point of need, including in the EHR.

**Intent.** Information technology role. Because KBI resources have made a substantial shift to online formats, it is imperative that the information technology team and the librarian communicate regularly regarding technology needs and changes, including but not limited to authentication tools, networking, systems, integration in the EHR, etc. Migrations, upgrades, and vendor-based changes can impact access to costly licensed resources that can hinder if not prevent access altogether. The librarian and the information technology team must work closely to maintain consistent access and may need to troubleshoot issues to ensure library users have access to all the organization's KBI resources while onsite using standard desktop hardware or at the bedside using a mobile device or working remotely [13, 21, 22].

**Standard 5:** Evidence provides the scientific basis linking KBI and improved patient care; enhanced patient education; support for performance improvement projects; patient safety functions; and student, medical, nursing, graduate and continuing medical education.

**Intent:** Patient care and safety. The librarian serves all user groups in the hospital and outlying clinics as applicable. The functions listed in Standard 2 are among the most dependent on evidence. Examples of connections and evidence include:

- **Patient care:** Frequent provision of evidenced-based information to support patient care decisions, integration of evidenced-based resources to support clinical and hospital staff providing information at the point of care, supporting morning rounds, and providing case-specific literature needed in support of rounds or other related activities [20, 23, 24].
- **Performance improvement and patient safety:** Active membership of the librarian on performance improvement committees and patient safety teams. Frequent provision of information on which performance improvement and patient safety decisions are based; routing current literature to appropriate individuals, committees, and teams relevant to the hospital's quality indicators, top chronic conditions/diagnoses, performance improvement projects, patient safety goals and/or identified problem areas [25].
- **Patient education:** Active membership of the librarian on patient education teams; consultation with team concerning selection, creation, and quality filtering of sources for patient education materials; provision of (or facilitation of access to) patient education materials for clinical staff that are diverse, culturally and age-appropriate and understandable; provision and marketing of library services directly to patients and families; and instruction in utilizing online resources for patient education [23].
- **Education of hospital's clinical, nursing, and medical staff:** Active membership of the librarian on teams or committees concerned with diversity, equity and inclusion initiatives and clinical, nursing and medical educational functions; provide regular input regarding online resources and materials to support planning and preparation of educational activities; education of hospital clinical, nursing, and medical staff in accessing, retrieving, searching online resources; instruction in appraising and evaluating evidence-based information and peer reviewed resources; support and encourage the use of technology, medical mobile applications and other resources to support education and training; identification of print and electronic resources to support individualized learning and educational development; participate in continuing medical education (CME), graduate medical education (GME), clinical nursing education (CNE) [26, 27].
- **Clinical education** (nursing) and resident/medical students; provision of specific literature to support grand rounds and related activities; and provision of access and instruction to virtual learning and other technology [28].

**Standard 6:** The librarian provides evidence of an ongoing assessment of the information needs of the organization and the development and implementation of a plan to provide appropriate resources, services, and technology to meet those identified needs.

**Intent.** Evaluations of KBI needs. The librarian uses a variety of tools and techniques, both formal and informal, direct and indirect, to assess the information needs of the hospital and medical staff. In response, resources and services are made available to meet those identified needs. Techniques may include, but are not limited to, focus groups, library committees, surveys, analysis of usage patterns, budget and strategic planning, inventory of collections, and one-on-one conversations with health care leaders regarding clinical and organizational information needs [29–33].

The librarian assures access to a current and authoritative collection of information that can be used for evidence-based practice, scholarly activity, and continuing education. Key resources, such as print, e-resources, multimedia, databases, etc., can be identified by review products such as Doody's Core Titles or by seeking recommendations from hospital staff or the national hospital library community. Resource-sharing agreements and membership in consortia should be utilized to enable efficient provision of additional resources.

Services should include expert literature searching, document delivery, and interlibrary loan. A recent benchmarking survey may be useful for determining what services other hospital libraries provide. Services should be ongoing and consider timeliness of delivery and user satisfaction [19].

Technology considerations include providing and maintaining a library catalog and/or discovery service to identify and locate library holdings; providing and maintaining a mechanism for remote access of library resources; and integration of library resources within EHR, intranet, and/or public website for easy access

A strategic plan can help a librarian analyze trends and anticipate future needs. A plan for providing information needs to take into account the values of the broader organization.

**Standard 7:** The library actively promotes KBI services and resources to all user groups and provides documented evidence thereof.

**Intent.** Service promotion. Promotion increases user awareness and efficient use of the services and resources available [34]. The library serves not only clinical staff, but may serve others, including:

- administrative and managerial staff,
- research staff,
- staff in off-site locations,
- students in affiliated programs,
- patients and their families, and
- other groups as applicable

Promotion of services may take the form of:

- announcements to hospital and/or medical staff of new services, resources, or offerings,
- inclusion of library services and resources in orientation of interns and residents (if applicable), nurses, and new medical and hospital staff members,
- activities in observance of National Library Week and/or National Medical Librarians Month,
- participation in information fairs, skills fairs, Research Day, or Match Day activities
- promotion of existing current awareness services or proactive provision of these services,
- plan for reaching library nonusers, and
- presentations to groups on what the library can offer them.

**Standard 8:** All KBI functions are performed in compliance with applicable federal, state, and local laws and regulations including but not limited to copyright, HIPAA (patient privacy and confidentiality), and vendor licensing agreements [35, 36].

**Intent.** Regulatory compliance. Interlibrary loan, document delivery, and onsite and remote access to subscription resources, as well as copying, faxing and printing of biomedical literature must adhere to vendor license agreements, publishing contracts, and federal and/or international copyright legislation. In addition, the hospital library adheres to the Health Information Portability and Accountability Act (HIPAA) and patient confidentiality when assisting patients, families, health care providers and others who may use the library or any of its services or resources [34, 35].

**Standard 9:** Service availability. KBI resources are available to clinical staff twenty-four hours a day, seven days a week.

**Intent.** Clinical decision-making occurs at all hours, so access to KBI must be continuously available. This is reinforced by the Joint Commission's element of performance for the Information Management standard IM.03.01.01: “The hospital provides access to knowledge-based information resources 24 hours a day, 7 days a week” [5, 7]. The Accreditation Council for Graduate Medical Education also requires that “Residents must have ready access to specialty-specific and other appropriate reference material in print or electronic format” (I.D.3.) [26].

Continuous access to resources may take multiple forms, depending on the size and structure of the institution. A broad selection of resources should be made available in a physical library and/or through electronic means. Physical resources should be made continually accessible to clinical staff, and the library should provide onsite and remote access to electronic resources.

**Standard 10:** The term *library* refers to the department—physical, virtual or a combination thereof—and includes a qualified librarian and support professionals, services, and resources that when put together, best meet the information needs of the health care organization.

**Intent.** Library as space. The term *library* has evolved and should reflect the dynamics of the health information world. Given the ever-changing needs of a health care organization, a library must be nimble and flex to meet the needs of users. Traditionally, the library was a physical space located onsite with a print collection. Today a library can be a single physical location that provides resources, staff, and services to one or more facilities, or it could be a completely virtual presence, or any combination of the two. A library that meets the needs of the organization it serves, whether physical, virtual, or a combination of the two is a library that is likely to be well-utilized.

In the case of a physical library, the space should be large enough to accommodate walk-in users and the library staff, any in-house collection(s), and an appropriate number of computer or other IT hardware workstations. Space should permit demonstration of and access to resources and ample seating for users. A separate office for staff ensures privacy of communication among library staff and confidentiality for persons requesting information [37].

**Standard 11:** IT resources are available to support the library's mission of providing KBI resources and services.

**Intent.** IT resources. Adequate IT resources are essential in the provision of up-to-date KBI resources and services. The library must have hardware and library-specific software applications to perform basic functions related to acquiring, organizing, retrieving, and delivering KBI resources to support the institution's mission. The library also must have internet connections with sufficient speed, performance, and bandwidth to access the many web-based resources now available [6, 7, 11–13, 21].

Hospitals have specific security and privacy issues. Therefore, the library director/manager should work in concert with the institution's IT department to assure that users' needs to access essential web-based KBI resources from the point of need are balanced with the network security needs and firewall policies of the institution. Examples of essential information technology resources include:

- access to the internet sufficient to use email, DOCLINE, OCLC, PubMed, and any commercial databases and full-text resources to which the library may subscribe,
- access should be convenient for all users in the library's institutions twenty-four hours, seven days a week; remote access should be available as licenses permit,
- specialized library software that can describe and track library resources and their use (e.g., catalog, circulation, serials control, and/or an integrated library system) appropriate to the library's collection and services; this software can be hosted locally or remotely, and
- access to high-bandwidth communication technologies (e.g., full-motion video, video streaming, and webcasting) appropriate to the library's services and its institution's educational programs.

## CONCLUSION

As the team worked, we acknowledged that we stood in the shadow of those who went before us. The original Hospital Libraries Section of MLA saw the need for cohesiveness in setting out a philosophy of services unique to those serving the medical profession. Standards established in response to those set forth by the medical associations forged a bond assuring physicians of easy access to authoritative literature. As the fields of medical sciences progressed, so did the need for updated hospital library standards.

The team also stood on the shoulders of our contemporaries. As we looked back and around, we recognized with professional pride how the term “health sciences librarians” easily expanded from serving physicians to including nurses, pharmacists, physical and allied health therapists, technicians, and ultimately administrators. As colleagues, we generously shared our time, wisdom, and experience with each other in revising the 2007 standards. We also accept that new research and knowledge will ultimately require continued and timely revisions.

The Task Force includes representatives from diverse fields with varying levels of experience. Our passion for using our talents to make the best and the latest knowledge available to our health care teams knows no bounds and aims for inclusivity. We offer the 2022 Standards as evidence of our commitment to patient care and safety and clinical education for all staff and patients.

## Data Availability

There is no data associated with this article.

## SUPPLEMENTAL FILES

**Appendix A:** Health Science Information Consortium of Toronto (HSICT)

**Appendix B:** Library systems

**Appendix C:** Health sciences library consultants

**Appendix D:** Glossary

## References

[1] Gluck JC, Hassig RA, Balogh L, Bandy M, Doyle JD, Kronenfeld MR, Lindner KL, Murray K, Petersen JA, Rand DC. Standards for hospital libraries 2002. J Med Libr Assoc. 2002 Oct;90(4):465–72. 12398254 PMC128964

[2] Hassig RA, Balogh L, Bandy M, Doyle JD, Gluck JC, Lindner KL, Reich B, Varner D. Standards for hospital libraries 2002 with 2004 revisions. J Med Libr Assoc. 2005 Apr;93(2):282–3. 15858633 PMC1082947

[3] Bandy M, Doyle JD, Fladger A, Frumento KS, Girouard L, Hayes S, Rourke D. Standards for hospital libraries 2007. J Med Libr Assoc. 2008 Apr;96(2):162-94. DOI: 10.3163/1536-5050.96.2.162. 18379675 PMC2268237

[4] Bandy MM, Dudden RF, eds. The Medical Library Association guide to managing health care libraries. Second edition. NY: Neal-Schuman; 2011.

[5] Joint Commission. Program: Hospital: Knowledge-based information resources are available, current and authoritative (IM.03.01.01) [Internet]. In Joint Commission Resources E-dition. Oakbrook, IL: The Commission; [cited 2021 Oct 27].

[6] Joint Commission. Program: Hospital: The hospital plans for continuity of its information management processes (IM.01.01.03) [Internet]. In Joint Commission Resources E-dition. Oakbrook, IL: The Commission; c2021 [cited 2021 Oct 27].

[7] Joint Commission. Program: Hospital: The hospital plans for managing information (IM.01.01.01) [Internet]. In Joint Commission Resources E-dition. c2021. Oakbrook, IL: The Commission; [cited 2021 Oct 27].

[8] Harnegie MP. Penetrating the hospital environment and promoting library resources. J Hosp Librariansh. 2015;15(2):217-225. 10.1080/15323269.2015.1014764.

[9] Medical Library Association. Partner with Hospital Librarians to improve patient care [Internet]. Chicago: The Association; [cited 2022 Mar 25]. https://www.mlanet.org/p/cm/ld/fid=1961.

[10] American Library Association. ALA Accredited Programs [Internet]. Chicago: The Association; [cited 1/20/2022]. https://www.ala.org/educationcareers/node/262/.

[11] Medical Library Association. Academy of health information professionals (AHIP) [Internet]. Chicago: The Association; [cited 2021 Apr 15]. https://www.mlanet.org/p/cm/ld/fid=41.

[12] Medical Library Association. Professional competencies [Internet]. Chicago: The Association; [cited 2021 Apr 15]. https://www.mlanet.org/p/cm/ld/fid=1217.

[13] Medical Library Association. Health information profession [Internet]. Chicago: The Association; c2021 [cited 2021 Apr 15]. https://www.mlanet.org/p/cm/ld/fid=53.

[14] Cooper ID, Crum JA. (2013) New activities and changing roles of health sciences librarians: a systematic review, 1990-2012. J Med Libr Assoc. 2013 Oct;101(4):268-77. DOI: 10.3163/1536-5050.101.4.008. 24163598 PMC3794682

[15] Morgan-Daniel J, Goodman XY, Franklin SG, Bartley K, Noe MN, Pionke JJ. Medical Library Association Diversity and Inclusion Task Force Report. Appendix D. J Med Libr Assoc. 2021 Jan 1;109(1):141-153. DOI: 10.5195/jmla.2021.1112 PMC7772976 33424477

[16] Frati F, Oja LA, Kleinberg J. CHLA Standards for Library and Information Services in Canadian Health & Social Services Institutions 2020. J Can Health Libr Assoc. 2021;42(1): 14-44. DOI: 10.29173/jchla29526. 35949504 PMC9327607

[17] Van Moorsel G. Analysis of compliance of hospital libraries with the Medical Library Association staffing standard: Examination of the current state of the industry and reconsideration of the standard. J Hosp Librariansh. 2009;9(3):273-285. DOI: 10.1080/15323260903030938.

[18] Health Science Information Consortium of Toronto. Library Value Toolkit. Benchmarking; Give me an example [Internet]. [cited 1/20/2022]. https://guides.hsict.library.utoronto.ca/c.php?g=430008&p=2932207.

[19] Spencer A, Mamo E, Billman BL. Benchmarking study of hospital libraries. MLA Hypothesis. 2021;31(1). DOI: 10.18060/23805.

[20] Brettle A, Maden M, Payne C. The impact of clinical librarian services on patients and health care organisations. Health Info Libr J. 2016 Jun;33(2):100-20. DOI: 10.1111/hir.12136. 26887653

[21] Matthews AP, Flake D. Everything you need in an electronic medical record (EMR): From patients to diagnoses to librarians, J Hosp Librariansh. 2016;16(2):97-106. DOI: 10.1080/15323269.2016.1150726.

[22] Hamasu C, Burroughs CM, Glenn E, Ball AL. Mining data in electronic health record systems: Opportunities for librarians. J Hosp Librariansh. 2017;17(4):282-291. DOI: 10.1080/15323269.2017.1366776.

[23] Sollenberger, J. F., & Holloway, R. G., Jr The evolving role and value of libraries and librarians in health care. JAMA. 2013 Sep 25; 310(12):1231–1232. DOI: 10.1001/jama.2013.277050 24065006

[24] Carlson R, Towner Wright S. Essential Services of Clinical Librarians in Academic and Health Care Settings: A Cross-Sectional Study. Med Ref Serv Q. 2021 Apr-Jun;40(2):168-187. DOI: 10.1080/02763869.2021.1912570. 33970819

[25] Saimbert, M. K., Zhang, Y., Pierce, J., Moncrief, E. S., O'Hagan, K. B., & Cole, P. Medical librarians supporting information systems project lifecycles toward improved patient safety. Medical librarians possess expertise to navigate various search resources and can investigate inquiries during IS project lifecycles. Journal of healthcare information management: JHIM. 2010 Winter;24(1):52–56. 20077926

[26] Accreditation Council for Graduate Medical Education. ACGME common program requirements (residency). [Internet]. Chicago: ACGME; c2000-2021 [cited 15 Apr 2021]. https://www.acgme.org/what-wedo/accreditation/common-program-requirements/.

[27] American Nurses Credentialing Center. Magnet model–creating a Magnet culture [Internet]. Chicago: American Nurses Association; [cited 15 Apr 2021]. https://www.nursingworld.org/organizational-programs/magnet/magnet-model/.

[28] National Committee for Quality Assurance. Physician and hospital quality certification [Internet]. Washington DC; NCQA; [cited 15 Apr 2021]. https://www.ncqa.org/programs/health-plans/physician-and-hospital-quality-phq/?utm_medium=cpc&utm_campaign=edu-smart-text&utm_source=google&utm_term=00000000.

[29] Jones DA, Esparza JM, Duggar DC. Initial entry of knowledge-based information (KBI) into the electronic health record and internal medicine residents' perceptions of KBI resources in the record, J Hosp Librariansh. 2011;11(4):325-337. DOI: 10.1080/15323269.2011.611108.

[30] Bleich MR, Brown R. Evidence-based leadership practice and the role of the librarian. J Contin Educ Nurs. 2019 Dec 1;50(12):537-539. doi: 10.3928/00220124-20191115-03. 31774923

[31] Oelschlegel S, Grabeel KL, Tester E, Heidel RE, Russomanno J. Librarians promoting changes in the health care delivery system through systematic assessment. Med Ref Serv Q. 2018 Apr-Jun;37(2):142-152. DOI: 10.1080/02763869.2018.1439216. 29558326

[32] Harnegie MP. Evaluation of library usage and attitudes of residents and fellows: Results of 017–2019 surveys, J Hosp Librariansh. 2021;21(2):141-157. DOI: 10.1080/15323269.2021.1899781

[33] Coleman DE, Robbins K. Strategic planning for a single person library. J Hosp Librariansh. 2016;16(4):299-304. J Hosp Librariansh. 2017;17(4):282-291.

[34] Delawska-Elliott B, Grinstead C, Martin HJ. Developing a Marketing Orientation in Hospital Library Services: A Case Report. Med Ref Serv Q. 2015;34(4):481-9. DOI: 10.1080/02763869.2015.1082390. 26496402

[35] Centers for Disease Control and Prevention. Health Insurance Portability and Accountability Act of 1996 (HIPAA). [Internet]. Atlanta, GA; CDC; rev 2018 [cited 17 Jan 2022]. https://www.cdc.gov/phlp/publications/topic/hipaa.html.

[36] U. S. Copyright Office. What is copyright? [Internet]. Washington DC; [cited 17 Jan 2022]. https://www.copyright.gov/what-is-copyright/.

[37] Gibson DS, Hernandez M, Draemel A. Destination library: Validating the importance of physical space. J Hosp Librariansh. 2015;15(3):251-261. DOI: 10.1080/15323269.2015.1049057.

[38] Case Western Reserve, University Archives. Glossary of Archival Terminology. [Internet]. Cleveland, OH: The University; c2007 [cited 17 Jan 2022]. https://case.edu/its/archives/Records/glossary.htm.

[39] Western New York Library Resources Council. Hospital Library resources program [Internet]. Cheektowaga, NY: The Council. C2022 [cited 17 Jan 2022]. https://www.wnylrc.org/hospital-library-services-program.

[40] Medical Library Association. Consumer health specialization [Internet]. Chicago, IL: The Association; c2021 [cited 2021 Apr 15]. https://www.mlanet.org/p/cm/ld/fid=329.

[41] HealthIT.gov. What is an electronic health record (EHR)? c2019 [cited 27 Oct 2021]. https://www.healthit.gov/faq/what-electronic-health-record-ehr.

[42] Indeed. What is a service level agreement? c2021 [cited 1Mar 2022}. https://www.indeed.com/career-advice/career-development/service-level-agreement.

