# Supplementary material for: Standards of practice for hospital libraries and librarians, 2022: Medical Library Association Hospital Libraries Caucus Standards Task Force
Source: J Med Libr Assoc. 2022 Oct 1;110(4):399–408. doi: 10.5195/jmla.2022.1590 (PMC10127117; doi:10.5195/jmla.2022.1590)
Supplement: Supplementary file 1 — Appendix A: Health Science Information Consortium of Toronto (HSICT) [file jmla-110-4-399-s01.pdf]

Internal use

Appendix A: Health Science Information Consortium of Toronto (HSICT)

	Levels of Library Service		
	Bronze	Silver	Gold
**Research**			
Literature searching	☐	☐	☐
Complex searches: systematic reviews			☐
Reference & citation support	☐	☐	☐
Consult on staff research strategies & grant applications (e.g. CIHR)			☐
Analysis of institutional or individual research impact metrics			☐
Embedded-Librarian support (e.g. clinical, labs, research)			☐
**Inter-Library Collaboration**			
Facilitate interlibrary loans/resource sharing	☐	☐	☐
Maximize efficiency via collaborative planning & purchasing		☐	☐
**Instruction and Teaching Support**			
Custom training on bibliographic tools (e.g. RefWorks, Endnote)		☐	☐
Develop & teach: Finding evidence-based practice resources		☐	☐
Develop e-learning videos			☐
Library orientation virtually/in-person	☐	☐	☐
Information literacy support & training		☐	☐
Custom resource training (databases, point of care resources etc.)	☐	☐	☐
**Customized Content Delivery / Push Services**			
Custom alerts, trackers & current awareness tools (RSS, TOC alerts)		☐	☐
Social media presence (Twitter, Facebook, etc.)			☐
Locate & deliver resources not available in collection		☐	☐
Review & market resources, incl. reports, websites, mobile apps, etc.	☐	☐	☐
Create health information apps			☐
**Collection Development**			
Identify, assess & negotiate contracts for new resources	☐	☐	☐
Resource analysis to ensure access to 'best possible' evidence	☐	☐	☐
Collaborate with consortia to maximize resource license discounts	☐	☐	☐
**Development and maintenance of search/discovery tools**			
Maintain intuitively searchable catalogue of library resources	☐	☐	☐
Provide a searchable interface for all library resources			☐
Develop & maintain library website	☐	☐	☐

Internal use

Enhance resources findability (link resolvers, discovery layers, etc.)			□
Develop resource guides (LibGuides) & curated web-portals			□
**Space/equipment management ***			
Provide independent study space with Wi-Fi	□	□	□
Provide group study space with Wi-Fi			□
Provide computer workstations & internet access	□	□	□
Ensure 24/7 access to computers		□	□
**Archives**			
Maintenance & collection of hospital archives			□
**Consumer Health Information**			
Provide consumer health information for patients & families		□	□
**Interprofessional Collaboration with Information Technology Services**			
Collaborate with IT to integrate resources into EMR			□
Collaborate with IT to maximize mobile device access to resources	□	□	□
Manage/curate large datasets ("big data")			□
**In-house Expertise**			
Support corporate information & knowledge management activities			□
Provide institutional support re copyright policies and procedures	□	□	□
Support continuing education for staff		□	□

https://guides.hsict.library.utoronto.ca/c.php?g=430008&p=2932207
