# Supplementary material for: Standards of practice for hospital libraries and librarians, 2022: Medical Library Association Hospital Libraries Caucus Standards Task Force
Source: J Med Libr Assoc. 2022 Oct 1;110(4):399–408. doi: 10.5195/jmla.2022.1590 (PMC10127117; doi:10.5195/jmla.2022.1590)
Supplement: Supplementary file 2 — Appendix B: Library systems [file jmla-110-4-399-s02.pdf]

Appendix B: Library systems

A library system can provide knowledge-based information (KBI) services and resources for its affiliates in several ways:

- Each affiliate hospital may maintain a separate library.
- Services and resources may be provided from a central location.
- Support staff may be present at each location, with professional services provided centrally.
- Support staff may be present at each location, with a circuit librarian arrangement.
- There may be a hybrid system, in which arrangements differ among affiliates.
- There may be coordinated resource sharing among the libraries.
- There may be a substantial network of electronic resources available to all affiliates.
- There may be virtual library services and resources provided.

These standards do not attempt to dictate a single manner in which services must be provided throughout a system. Rather, they provide a standard for the amount and nature of services and staffing that must be available for the system as a whole.

Depending on a host of factors, including physical proximity of the affiliates and the extent of electronic access to resources, the librarian and health system administration will collaborate in making decisions about centralization or decentralization of library resources and services and extent of staffing in the libraries. The committee strongly recommends that each location at least have professional and/or support staff, so that users may obtain assistance in finding needed information and the smooth flow of operations can be ensured. The amount of staffing—both professional and support level—throughout the system must be at least at the level specified in the library staffing formula, taking all components of the health care system into account.

Whether each hospital is treated separately in determining staffing levels or the system is taken as a whole is left to the judgment of the librarian and administrators. The important point is that staffing is sufficient to serve the number of users.If a decision is made to maintain and staff libraries at multiple locations, a level of staffing above that specified in the library staffing formula will be necessary to the extent that

- tasks will be duplicated and
- the time of the staff will be used to travel among institutions.

Each separate library location should have convenient, reliable access to a quality core collection of KBI resources, tailored to the needs of the institution. Whether this is provided as an in-house print collection, reliable electronic collection or a combination of both is a decision that may be made locally. An arrangement whereby core materials are physically housed in a central location and transmitted to other locations on demand (by fax or other means) is not an option, as this would preclude access to information needed for patient care during hours when the library is not staffed. This may, however, be a viable option for materials over and above the core collection. Technological infrastructure needs to be in place to support virtual library services. Appropriate equipment may include and is not limited to: desktop computers or laptops/notebooks, mobile phones, team collaboration software, secured network, virtual privacy network (VPN) or remote desktop protocol (RDP), etc.
