# Supplementary material for: Standards of practice for hospital libraries and librarians, 2022: Medical Library Association Hospital Libraries Caucus Standards Task Force
Source: J Med Libr Assoc. 2022 Oct 1;110(4):399–408. doi: 10.5195/jmla.2022.1590 (PMC10127117; doi:10.5195/jmla.2022.1590)
Supplement: Supplementary file 3 — Appendix C: Health sciences library consultants [file jmla-110-4-399-s03.pdf]

Appendix C: Health sciences library consultants

Role statement for health sciences library consultants. The health sciences library consultant provides advisory services to hospital library leaders, including library staff and/or a library committee, medical staff, and hospital administration.

These consultative services aid health sciences library leaders in defining the scope of library services, designing health sciences library services and in designing facilities to best meet the informational, educational, research, and patient care-related needs of the entire hospital community including, where appropriate, the instructional needs of patients.

Consultation is provided on a contractual basis. Hiring a hospital library consultant is not a substitute for the employment of permanent library personnel.

Qualifications for health sciences library consultants. A health science library consultant should meet all of the criteria listed in Standard 2 of this Hospital Library Standards of Practice document. In addition, the consultant should be a member of the Medical Library Association and its Academy of Health Information Professionals. The consultant should have at least three years of experience managing hospital libraries and be able to demonstrate a high level of knowledge in hospital library operational and service trends.
