# Supplementary material for: Standards of practice for hospital libraries and librarians, 2022: Medical Library Association Hospital Libraries Caucus Standards Task Force
Source: J Med Libr Assoc. 2022 Oct 1;110(4):399–408. doi: 10.5195/jmla.2022.1590 (PMC10127117; doi:10.5195/jmla.2022.1590)
Supplement: Supplementary file 4 — Appendix D: Glossary [file jmla-110-4-399-s04.pdf]

Appendix D: Glossary

Academy of Health Information Professionals (AHIP): the Medical Library Association's peer-reviewed professional development and career recognition program [13].

American Library Association (ALA): Founded in 1876, the oldest and largest library association in the world. ALA accredits master's programs in library and information studies across the United States, Canada, and Puerto Rico. Accreditation is achieved through a review process conducted by an external review panel of practitioners and academics that verifies that the program meets the *Standards for Accreditation of Master's Programs in Library and Information Studies* [10].

Archival management: Maintenance of an organization's non-current records, controlled by provenance, and retained for their continuing value in providing 1) evidence of the activities of the organization, or 2) information about entities affected by the organization; this may include physical storage and/or scanning and photographing of items for digital storage [38].

Canadian Health Libraries Association (CHLA): Established in 1976, the Association is a community of Canadian Health Library and Information professionals. The staffing formula in Standard 3 was adapted from the *CHLA Standards for Library and Information Services in Canadian Health & Social Services Institutions 2020* [16].

Circuit librarian: A librarian who provides service to two or more institutions, who regularly schedules on-site visits and provides off-site services when requested [39].

Clinical outreach: Typically consists of attending rounds with a health care team and providing case-specific information to assist with patient care.

Consumer health services: May include reference, education, and outreach for patients and/or other laypersons to provide information on health, medical conditions, or medical topics [40].

Core collection: Those comprehensive, authoritative print or electronic works to which users refer most often. The core collection should be tailored to the needs of a particular institution and can be expected to contain information pertinent to most reference queries. It may be supplemented by other resources.

Current awareness services: Services designed to keep users up to date on new developments and resources in their field of interest, including table of contents delivery, email alerts, etc.

DOCLINE: National Library of Medicine's interlibrary loan (ILL) request routing system. It supports resource sharing among participating libraries by enabling them to borrow from and lend to one another.

Electronic health record (EHR): digital version of a patient's paper chart. EHRs are real-time, patient-centered records that make information available instantly and securely to authorized users. May also be known as an electronic medical record (EMR) [41].

Embedded library services: Librarians may be embedded on the health care team by participating in rounds to provide information relevant to patient care, or they may be embedded virtually at the point of care by having service requests integrated into the electronic health record.

Evidence-based medicine (EBM): Conscientious, explicit, judicious, and reasonable use of modern, best evidence in making decisions about the care of individual patients; it represents integration of clinical expertise, patient's values and best available evidence in the process of decision making related to patient's health care.

Hospital library: A library that provides services and resources to hospital staff and/or patients. It may reside within the hospital or an affiliated institution or may be completely virtual. It may

also be referred to as a medical library, nursing library, health sciences library, biomedical library, consumer health library, etc.

Information technology team: The staff directing the hardware, software, network, security and related functions of the hospital or health care system.

Knowledge-based information (KBI): A collection of stored facts, models, and information that can be used for ongoing staff development, for designing and redesigning processes, and for solving problems. This includes medication-related information such as information regarding drug interactions, drug therapy, side effects, toxicology, dosage, indications for use, and routes of administration. Knowledge-based information is found in the clinical, scientific, and management literature.

Library consultant: Temporary advisor to the hospital administration, medical staff, library staff, and/or library committee in defining and designing hospital library services and/or facilities to meet the informational, educational, research, and patient care-related needs of the entire hospital community.

License agreement: A written contract between a library and an information provider that sets out the terms and conditions under which library users can use digital content.

Mediated literature search: Search of the literature performed by a librarian on behalf of a library user.

Medical Library Association (MLA): A global, nonprofit educational organization, since 1898, with a membership of more than 400 institutions and 3,000 professionals in the health information field, that has fostered excellence in the professional practice and leadership of health sciences library and information professionals to enhance health care, education, and research throughout the world.

Needs assessment: A systematic process designed to determine the need for specific services or types of information by the library's user groups. This may take the form of surveys, focus groups, interviews of groups or individuals, or other methods.

OCLC: A global library cooperative that provides shared technology services, original research and community programs for its membership and the library community at large. Many libraries use OCLC's WorldShare Interlibrary Loan system and EZProxy for remote access.

Patient education: The provision of information to help inpatients or outpatients, or their family members, understand and cope with the condition for which they are receiving medical care. This education assists patients and/or their families in taking an active role in health care decision making.

Performance improvement: The systematic process of detecting and analyzing performance problems, designing and developing interventions to address the problems, implementing the interventions, evaluating the results, and sustaining improvement.

Research data management (RDM): An emerging discipline where hospital libraries have staff that are trained to help users make a plan to develop, describe, protect, share, and preserve their data. Some libraries also maintain a data repository.

Service level agreement: A contract or agreement between the library and the hospital administration, which outlines performance expectations, availability requirements, key processes, and resolution for when issues may arise, which will guide departmental resource allocation [42].
